# BPC 157, L-NAME, L-Arginine, NO-Relation, in the Suited Rat Ketamine Models Resembling “Negative-Like” Symptoms of Schizophrenia

**DOI:** 10.3390/biomedicines10071462

**Published:** 2022-06-21

**Authors:** Andrea Zemba Cilic, Mladen Zemba, Matija Cilic, Sanja Strbe, Spomenko Ilic, Jaksa Vukojevic, Zoran Zoricic, Igor Filipcic, Antonio Kokot, Ivan Maria Smoday, Iva Rukavina, Alenka Boban Blagaic, Ante Tvrdeic, Bozidar Duplancic, Vasilije Stambolija, Darko Marcinko, Anita Skrtic, Sven Seiwerth, Predrag Sikiric

**Affiliations:** 1 Department of Pharmacology, School of Medicine, University of Zagreb, 10000 Zagreb, Croatia; azemba@gmail.com (A.Z.C.); mladen.zemba@gmail.com (M.Z.); matijacilic@hotmail.com (M.C.); silic67@gmail.com (S.I.); jaksavukojevic@gmail.com (J.V.); ivansmoday1@gmail.com (I.M.S.); iva_rukavina@hotmail.com (I.R.); abblagaic@mef.hr (A.B.B.); ante.tvrdeic@mef.hr (A.T.); 2 Department of Psychiatry, University of Zagreb School of Medicine, University Clinical Centre Zagreb, 10000 Zagreb, Croatia; strbes@gmail.com (S.S.); igor.filipcic@pbsvi.hr (I.F.); predstojnik.psi@kbc-zagreb.hr (D.M.); 3 University Department of Psychiatry, University Hospital Sestre Milosrdnice, 10000 Zagreb, Croatia; zoran.zoricic@kbcsm.hr; 4 Department of Anatomy and Neuroscience, Faculty of Medicine, J.J. Strossmayer University of Osijek, 31000 Osijek, Croatia; antonio.kokot@mefos.hr; 5 Department of Anesthesia, School of Medicine, 21000 Split, Croatia; bozidarduplancic@gmail.com; 6 Department of Anesthesiology, Resuscitation and Intensive Care, University Hospital Centre Zagreb, 10000 Zagreb, Croatia; vasilije.stambolija@gmail.com; 7 Department of Pathology, School of Medicine, University of Zagreb, 10000 Zagreb, Croatia; sven.seiwerth@mef.hr

**Keywords:** pentadecapeptide BPC 157, L-NAME, L-arginine, resembling “negative-like” schizophrenia symptoms, cognition dysfunction, social withdrawal, anhedonia, anxiogenicity, gene, rats

## Abstract

We attempted throughout the NO-system to achieve the particular counteraction of the ketamine-induced resembling “negative-like” schizophrenia symptoms in rats using pentadecapeptide BPC 157, and NO-agents, NG-nitro-L-arginine methylester (L-NAME), and/or L-arginine, triple application. This might be the find out the NO-system organized therapy (i.e., simultaneously implied NO-system blockade (L-NAME) vs. NO-system over-stimulation (L-arginine) vs. NO-system immobilization (L-NAME+L-arginine)). The ketamine regimen (intraperitoneally/kg) included: 3 mg (cognitive dysfunction, novel object recognition test), 30 mg (anxiogenic effect (open field test) and anhedonia (sucrose test)), and 8 mg/3 days (social withdrawal). Medication (mg/kg intraperitoneally) was L-NAME (5), L-arginine (100), and BPC 157 (0.01), alone and/or together, given immediately before ketamine (L-NAME, L-arginine, and combination) or given immediately after (BPC 157 and combinations). BPC 157 counteracted ketamine-cognition dysfunction, social withdrawal, and anhedonia, and exerted additional anxiolytic effect. L-NAME (antagonization, social withdrawal) and L-arginine (antagonization, cognitive dysfunction, anhedonia) both included worsening cognitive dysfunction, anhedonia, and anxiogenic effect (L-NAME), social withdrawal, and anxiogenic effect (L-arginine). Thus, ketamine-induced resembling “negative-like” schizophrenia symptoms were “L-NAME non-responsive, L-arginine responsive” (cognition dysfunction), “L-NAME responsive, L-arginine non-responsive” (social withdrawal), “L-NAME responsive, L-arginine responsive, opposite effect” (anhedonia) and “L-NAME responsive, L-arginine responsive, parallel effect” (both anxiogening). In cognition dysfunction, BPC 157 overwhelmed NO-agents effects. The mRNA expression studies in brain tissue evidenced considerable overlapping of gene overexpression in healthy rats treated with ketamine or BPC 157. With the BPC 157 therapy applied immediately after ketamine, the effect on *Nos1*, *Nos2*, *Plcg1*, *Prkcg*, and *Ptgs2* (increased or decreased expression), appeared as a timely specific BPC 157 effect on ketamine-specific targets.

## 1. Introduction

The applicability of the important NO abnormalities in the brain associated with schizophrenia [1] might be questioned with both excess and low nitric oxide (NO) levels linked to this pathology (although the direction of abnormalities is still under debate) [2], as well as with both NO-donors and NOS-inhibitors reviewed [3,4]. 

Thus, a new practical attempt, using the advantage of ketamine as a noncompetitive NMDA antagonist [5,6], should achieve throughout the NO-system the particular counteraction of the resembling “negative-like” schizophrenia symptoms. This ketamine rat study of the stable pentadecapeptide BPC 157, NO-agents, NG-nitro-L-arginine methylester (L-NAME) and L-arginine (for review, see [7,8,9,10,11]), by revealing the NO-system impediments and advantages, also might contend with the present lack of the therapy evidence of the primary negative schizophrenia symptoms [5]. Blunted affect, alogia (reduction in quantity of words spoken), avolition (reduced goal-directed activity due to decreased motivation), asociality, and anhedonia (reduced experience of pleasure (for review, see [5]), generally do not respond well to currently available treatment [5]. 

Thereby, these negative symptoms, approached in the noncompetitive NMDA antagonist ketamine-induced resembling “negative-like“ schizophrenia-like symptoms (cognitive dysfunction (novel object recognition test)) [12,13,14], social withdrawal [15,16], anhedonia (sucrose test) [17,18], anxiogenic effect (open field test) [18,19,20,21], clearly constitute an unmet medical need in schizophrenia [5]. This might be the opportunity to find out the NO-system organized therapy (i.e., simultaneously implied NO-system blockade (L-NAME) vs. NO-system over-stimulation (L-arginine) vs. NO-system immobilization (L-NAME+L-arginine)), that new and effective treatments from the stable pentadecapeptide BPC 157, NO-agents, L-NAME and L-arginine are needed. This combined approach (BPC 157, L-NAME and L-arginine, given alone and/or together in the suited rat models) by the particular therapy responses to L-NAME, L-arginine, and L-NAME and L-arginine, was already operational in the “positive-like” schizophrenia models [8]. There were defining the effective therapy (BPC 157 > L-arginine > L-NAME antagonizing potential) and the distinctive NO-system background (i.e., responsive to L-NAME or to L-arginine, to none or to both) of the “positive-like” schizophrenia models’ symptoms [8]. Distinction of the acute amphetamine, acute apomorphine and MK-801, chronic methamphetamine and acute haloperidol, as particularly related to NO-system, inhibition and/or stimulation, provided the amelioration throughout two responses: “L-NAME responsive, L-arginine responsive”, and “L-NAME non-responsive, L-arginine responsive”. Furthermore, the evidence in clarification and resolving of the dopamine and glutamate schizophrenia models [8] might be instructive. The stable gastric pentadecapeptide BPC 157, NO-agents, L-NAME, NO-synthase (NOS)-blocker, and L-arginine, NOS-substrate, given alone or together (triple application), known to particularly mutually interact and form particular NO-system connections (for review, see [8,9,10,11]) might result with the particular counteraction of each of the resembling “negative-like” schizophrenia symptoms (i.e., with L-NAME or L-arginine, with both or none). Likewise, based on its interplay with L-NAME, and L-arginine, and combination, each of the resembling the “negative-like” schizophrenia symptoms might be defined as related to NOS-blockade or to NOS-stimulation or NO-system not related.

The stable gastric pentadecapeptide BPC 157 was recently reviewed from the viewpoint of the cytoprotection concept (i.e., safe in ulcerative colitis trial, lethal dose (LD1) not achieved in toxicology studies, for review, see [7]), but its modulatory role in the NO-system and dopamine-system as central nervous system beneficial effects (for review, see [7,8,9,10,11]) also might approach the issue of the negative symptom of schizophrenia [5]. BPC 157 therapy, which counteracted the NO-system inhibition (including also L-NAME-induced catalepsy) and over-stimulation (L-arginine-induced adverse effects), and might induce NO-release by its own (for review, see [7,8,11]), claimed that it might control essential NO-molecular pathways [22,23]. In addition, it might counteract the dopamine inhibition (i.e., haloperidol-induced catalepsy), destruction and depletion (i.e., motor abnormalities induced by parkinsongenic neurotoxin 1-methyl-4-phenyl-1,2,3,6-tetrahydropyridine (MPTP) or reserpine) and over-stimulation (i.e., the adverse effects of the acute and chronic amphetamine, acute apomorphine, chronic methamphetamine) (for review, see [8,9,10]). In a specific manner, it might affect serotonin release in several brain areas, especially in substantia nigra [24]. In addition, as it was implicated for the NO-agents [25,26,27,28], BPC 157 has particular anxiolytic, anticonvulsant, and anti-depressant activity (for review, see [9,10]).

Importantly, this triple application, L-NAME vs. L-arginine vs. combination [8], would markedly overwhelm the regular level in the corresponding NO-studies, the application of the one single NO-agent (i.e., L-NAME) (i.e., for review, see [4]). Evidently, as demonstrated with the “positive-like” schizophrenia models’ symptoms [8], the triple application would better define the whole NO-system complexity (otherwise, the successful use of either NOS-blocker [12,29,30] or L-arginine [31,32] might disable each other value in the schizophrenia therapy [12,29,30,31,32]). This might be the case despite the fact that the neurobiology of the positive symptoms (i.e., mesolimbic pathways that involve dopamine and glutamate networks) is distinct from that of negative symptoms (i.e., fronto-cortical temporal and cortico-striatal pathways) [4,5]. In particular, positive symptoms of schizophrenia are associated with an excess of dopaminergic neurotransmission, in striatal brain regions, while negative symptoms and cognitive deficits are linked to dopaminergic hypofunction in prefrontal brain regions [4,5]. On the other hand, we might speculate that the particular modulatory role in the NO- and dopamine-system might be useful for the BPC 157 effectiveness as well (for review, see [7,8,9,10,11]). Thus, the separate (L-NAME or L-arginine) and combined (L-NAME+L-arginine) application of NO-agents [8], the implementation of all NO-function possibilities, and identification of the right one, for the each of the investigated negative-like symptoms items (L-NAME responsive or not, L-arginine responsive or not, mutual antagonization or not) might result with the demonstration of the multimodal NO-axis impact. NO-axis, able to react depending on the condition and the given NO-agent(s) [8] on the symptoms distinctively related to “negative-like” symptoms in the NMDA-receptor antagonist ketamine-induced schizophrenia rat model [12,13,14,15,16,17,18,19,20,21], might be a novel point. These similar or distinctive effects might suggest the “negative-like” symptoms to be particular, more or less closely related to each other. For the given agents, these similar or distinctive effects might suggest a particular agent’s effect, depending on its NO-modulatory ability, either able to affect all symptoms in the same way (i.e., BPC 157), or to ameliorate some but worsen others (i.e., L-arginine and L-NAME) [8].

Thereby, in the ketamine-rats, we applied BPC 157, L-NAME and L-arginine alone and/or combined, as triple application. Note, as mentioned, that the triple application that would cover all NO-system functioning, blockade (L-NAME), over-stimulation (L-arginine), and immobilization (L-NAME+L-arginine) [8,9,10,11] was regularly not used in corresponding studies with NO-agent application (i.e., for review, see [4]). The assessment was carried out in the ketamine-induced resembling “negative-like “schizophrenia-like symptoms (cognitive dysfunction (novel object recognition test)) [12,13,14], social withdrawal [15,16], anhedonia (sucrose test) [17,18], and anxiogenic effect (open field test) [18,19,20,21]. In addition, to minimize at least partly the limitations known in the prime behavioral studies and to highlight a likely special point to explain how the dysfunction and its counteraction is causal to or the result of ketamine/BPC 157 interactions, a particular gene expression was carried out in the brain, providing their particular association with schizophrenia conditions. Analyzed [33] were *Nos1* [34,35], *Nos2* [36,37], *Nos3* [38], phospholipase C, gamma 1 (*Plcg1*) [39,40], protein kinase C gamma (*Prkcg*) [41,42], prostaglandin-endoperoxide synthase 2, cyclooxygenase (Cox)2 (*Ptgs2*) [43], and protein tyrosine kinase 2 (*Ptk2*) [44,45].

## 2. Materials and Methods

### 2.1. Animals

Male Wistar rats weighing 200 g–250 g randomly assigned (6 rats per group) by the observers unaware of the treatment. Animals were housed 2/cage with bedding, in a room with controlled temperature (23 ± 1 °C) and a 12-h light/dark cycle. Water and standard rodent chaw were continuously available in the home cage. Procedures approved by the Local Committee (643-03-01-19101/1, 380-59-10106-19-1302/3), were carried out in accordance with the Guide for the Care and Use of Laboratory Animals and Directive 2010/63/EU and assessment carried out at 2 p.m. in transparent cage, in a soundproof, light, temperature-controlled room, by the observers unaware of the treatment.

### 2.2. Drugs

As previously described [8], the medication without carrier or peptidase inhibitor included stable gastric pentadecapeptide BPC 157 (a partial sequence of the human gastric juice protein BPC, freely soluble in water at pH 7.0 and in saline). It was prepared as a peptide with 99% (HPLC) purity (1-des-Gly peptide was the main impurity; manufactured by Diagen, Ljubljana, Slovenia, GEPPPGKPADDAGLV, M.W. 1419) (in dose and application regimens as described before [11]. The peptide with 99% high-pressure liquid chromatography (HPLC) purity, 1-des-Gly peptide as a biologically inactive impurity, was used [4]. L-NAME, L-arginine were commercially purchased (Sigma-Aldrich, St. Louis, MO, USA), ketamine (ketamine hydrochloride, Richter Pharma, Wels, Austria) (cognitive impairment, social withdrawal), ketamine S (+), Pfizer, New York, NY, USA (anxiety and anhedonia) were used.

### 2.3. Novel Object Recognition Test

Before testing, for 3 consecutive days, rats were allowed to explore the apparatus for 2 min [12,13]. Testing consisted of a session of two 2-min trials. *“Sample” trial* (*T1*). In two opposite corners of the apparatus in a random manner, two identical samples (objects) were placed. A rat was placed in the middle of the apparatus and was left to explore these two identical objects. *Application*. Immediately after T1, the application protocol (post-training memory components (storage and/or retrieval)) was carried out as follows. In the ketamine rats (3 mg/kg ip) (dose adjusted accordingly to [12,14]), medication (mg/kg ip) was: L-NAME (5), L-arginine (100), BPC 157 (0.01), alone and/or together (L-NAME+L-arginine, L-arginine+BPC 157, L-NAME+BPC 157, L-arginine+L-NAME+BPC 157), and saline 5 mL/kg ip (control ketamine). The application was immediately before ketamine (L-NAME, L-arginine, and combination L-NAME+L-arginine) or the application was immediately after ketamine (BPC 157 and combinations L-NAME+BPC 157, L-arginine+BPC 157, L-NAME+L-arginine+BPC 157). *Resting period*. After T1, the rat was put back in its home cage and a 1 h intertrial interval (ITI) was given. “*Choice*” *trial* (*T2*). At the end of the resting (intertrial interval) period, the “choice” trial (T2) was performed. During T2, a new object (N) replaced one of the samples presented in T1, therefore, the rats were re-exposed to two objects: the familiar (F) and the new (N). *Assessment*. The times spent by rats in exploring each object during T1 and T2 were recorded manually by using a stopwatch. Exploration was defined directing the nose to the object at a distance of not more than 2 cm and/or touching the object with the nose. From this measure a series of variables was then calculated: the total time spent in exploring the two identical objects in T1, and that spent in exploring the two objects (F) and (N) in T2. The discrimination between (F) and (N) during T2 was measured by comparing the time spent in exploring the (F) with that spent in exploring the (N). As this time may be biased by differences in overall levels of exploration a discrimination index (D) was then calculated; D = N − F/N + F. D is discrimination ratio and represents the difference in exploration time expressed as a proportion of the total time spent exploring the two objects in T2.

### 2.4. Social Interaction Test

The experiment included the ketamine rats (8 mg/kg ip throughout 3 days) and was performed in an open arena (length, width, height: 150 × 100 × 40 cm), as described by Koros et al. [15]. On the day of testing, rats previously color-coded and without contact with each other received identical treatment and were simultaneously placed in opposite corners of the arena. In the ketamine rats (8 mg/kg ip throughout 3 days), medication (mg/kg ip) was with the last ketamine application: L-NAME (5), L-arginine (100), BPC 157 (0.01), alone and/or together (L-NAME+L-arginine, L-arginine+BPC 157, L-NAME+BPC 157, L-arginine+L-NAME+BPC 157), and saline 5 mL/kg ip (control ketamine). The application was immediately before ketamine (L-NAME, L-arginine, and combination L-NAME+L-arginine) or the application was immediately after ketamine (BPC 157 and combinations L-NAME+BPC 157, L-arginine+BPC 157, L-NAME+L-arginine+BPC 157). After 30 min, the rats behavior was recorded on camera for 10 min. Social behavior measured for each rat in the pair rated as duration of social interaction included: sniffing, grooming, tracking, hitting, climbing, jumping, wrestling/boxing, and crawling below/above, partner [16]. The time spent by each rat in the pair in the behaviors described above was summarized into a unique assessment of social interaction. In addition, locomotor activity was recorded as the total number of steps of each rat in the pair during the 10 min observation. In order to erase olfactory traces, the arena was cleaned with 20% ethanol after each experiment and dried on paper.

### 2.5. Open Field Test

In the ketamine rats (30 mg/kg ip), medication (mg/kg ip) was: L-NAME (5), L-arginine (100), BPC 157 (0.01), alone and/or together (L-NAME+L-arginine, L-arginine+BPC 157, L-NAME+BPC 157, L-arginine+L-NAME+BPC 157), and saline 5 mL/kg ip (control ketamine). The application was immediately before ketamine (L-NAME, L-arginine, and combination L-NAME+L-arginine) or the application was immediately after ketamine (BPC 157 and combinations L-NAME+BPC 157, L-arginine+BPC 157, L-NAME+L-arginine+BPC 157). After 30 min, the locomotor activity of rats was first recorded by a camera for 20 min inside an open field arena with a white floor bounded by walls measuring 46 × 46 cm. The open field was divided by black lines into 64 squares measuring 5.75 × 5.75 cm. The central 16 squares were defined as the central zone, in which animal activity was assessed as a measure of anxiety-like behavior [19]. The test was performed as previously described [18,19,20]. Rats were tested only once in open field, and were initially placed in the center of the field. The variables observed were: (a) total number of trajectories (i.e., horizontal activity); (b) number of trajectories in the central open field zone; (c) number of ascents (i.e., vertical activity). Data were recorded individually, and values were displayed as an average in 4 min blocks or as a total score in 20 min. Changes in the pattern of locomotion in the open field, such as hyperactivity (increased vertical and horizontal activity) are usually interpreted as psychotic behaviors. Anxiety-like behavioral measures included the relative proportion of trajectories conducted in the study of central squares relative to those located along the arena walls [20]. To avoid the presence of odor signs, the open field arena was thoroughly cleaned with 20% ethanol and then cleaned with dry paper after each experiment.

### 2.6. Anhedonia (Sucrose Preference Test)

After the end of the open field test and assessment of medication (mg/kg ip) effect (L-NAME (5), L-arginine (100), BPC 157 (0.01), alone and/or together (L-NAME+L-arginine, L-arginine+BPC 157, L-NAME+BPC 157, L-arginine+L-NAME+BPC 157), and saline 5 mL/kg ip (control ketamine); application immediately before ketamine (L-NAME, L-arginine, and combination L-NAME+L-arginine) or application immediately after ketamine (BPC 157 and combinations L-arginine+BPC 157, L-NAME+BPC 157, L-arginine+L-NAME+BPC 157) was carried out, sucrose preference test was examined by placing one animal per cage for 72 h with free access to food and with two identical graduated water bottles randomly allocated in the cage (changed after each 24 h), one containing 250 mL of top water and the other 250 mL pf 1% *w*/*v* sucrose (Sigma-Aldrich, St. Louis, MO, USA) in tap water [18]. The final volume of each bottle was measured at the end of 24 h, during 3 consecutive days. Thus, sucrose reference was calculated as the ratio of sucrose intake to total fluid intake and values converted to percentage [18].

### 2.7. Gene Expression Analysis

Healthy rats were treated with ketamine (30 mg/kg ip) or BPC 157 (10 ng/kg ip). The other rats received ketamine (30 mg/kg ip) and immediately thereafter saline (5 mL/kg ip) or ketamine (30 mg/kg ip) and immediately thereafter BPC 157 (10 ng/kg ip). Rats were sacrificed at 5 min, 30 min, and 60 min after medication and the brain tissue was rapidly harvested by dissection and snap-frozen in liquid nitrogen.

Samples were homogenized using the Bio-Gen PRO200 homogenizer (PRO Scientific, Oxford, CT, USA) in 1000 μL of TRIzol (Invitrogen, Thermo Fisher Scientific, Waltham, MA, USA) and total RNA extraction was performed according to manufacturer’s instructions using a TRIzol-based reagent method.

RNA quantification was done using the DeNovix DS-11 Spectrophotometer (DeNovix Inc., Wilmington, DE, USA). Results were used to perform reverse transcription with High Capacity cDNA Reverse Transcription Kit (Applied Biosystems, Thermo Fisher Scientific, Waltham, MA, USA). Manufacturer’s instructions were followed and GeneAmp PCR System 9700 machine (Applied Biosystems, Thermo Fisher Scientific, Waltham, MA, USA) was used.

RT-qPCR analysis was carried out using the Cobas z 480 instrument (Hoffmann-La Roche Ltd., Basel, Switzerland) with TaqMan Gene Expression Master Mix (Applied Biosystems, Thermo Fisher Scientific, Waltham, MA, USA) and specific TaqMan Gene Expression Assays (Applied Biosystems, Thermo Fisher Scientific, Waltham, MA, USA) for housekeeping gene *Gapdh* (Assay ID: Rn01775763_g1) and targeted genes: *Nos1* (Assay ID: Rn00583793_m1), *Nos2* (Assay ID: Rn00561646_m1), *Nos3* (Assay ID: Rn02132634_s1), *Plgc1* (Assay ID: Rn00566108_m1), *Prkcg* (Assay ID: Rn00440861_m1), *Ptgs2* (Assay ID: Rn01483828_m1) and *Ptk2* (Assay ID: Rn01505115_m1). Quantitative PCR was carried out in duplicate for every sample under the following thermal cycling conditions: 2 min at 50 °C, 10 min at 95 °C, 45 cycles of 15 s at 95 °C and 1 min at 60 °C.

Gene expression differences between treated and non-treated samples were analyzed using the formula 2^−ΔΔCt^, where ΔΔCt is the difference between ΔCt of treated sample and ΔCt of non-treated sample. Results were expressed as fold change. Fold change values < 1.00 indicates decreased gene expression in treated animals (downregulation), and fold change values > 1.00 indicated increased gene expression in treated animals (upregulation).

### 2.8. Statistical Analysis

Data analysis and charting were performed using Kyplot version 6 (https://www.kyenslab.com/en-us/about-kyplot-6/, accessed on 1 June 2022). GraphPadPrism version 5 (https://www.graphpad.com/sciaching-software/prism/, accessed on 1 June 2022). All applied tests were bidirectional, and *p* ≤ 0.05 values were considered statistically significant [46]. A comparison of the two groups was performed using the Mann–Whitney test. Comparison of multiple groups with the control group was performed by Dunnett’s multiple comparison test [46].

## 3. Results

### 3.1. Novel Object Recognition Test, Cognitive Dysfunction

**Figure 1:**
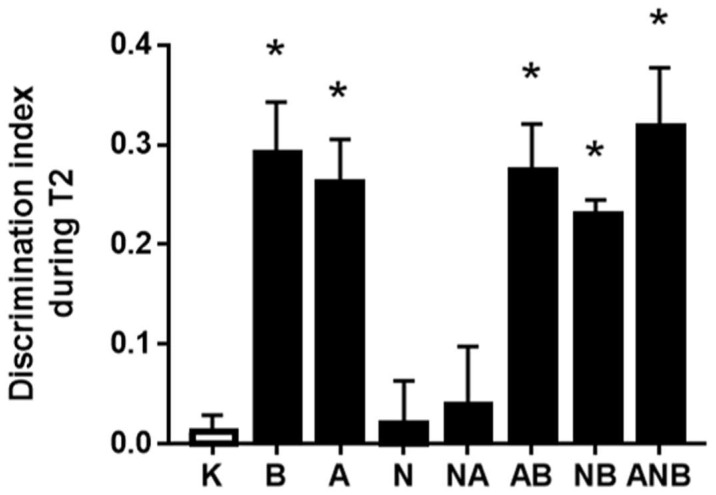
Discrimination index D expressed by different groups of rats during T2. The discrimination index D in the novel object recognition test for ketamine-induced disorder in T2, mean values, and 95 percent confidence intervals are presented. The application protocol was injected ip immediately after T1. 1 h ITI was used. In ketamine rats (3 mg/kg ip), medication (mg/kg ip) was: L-NAME (5) (N), L-arginine (100) (A), BPC 157 (0.01) (B), alone and/or together (L-NAME+L-arginine (NA), L-arginine+BPC 157 (AB), L-NAME+BPC 157 (NB), L-arginine+L-NAME+BPC 157 (ANB)), and saline 5 mL/kg ip (control ketamine) (K). The application was immediately before ketamine (L-NAME, L-arginine, and combination) or the application was immediately after ketamine (BPC 157 and combinations). Dunnett’s multiple comparison test was used to compare control group (K) with treated groups. * *p* < 0.05 relative to ketamine.

Acute ketamine treatment (3.0 mg/kg ip) caused a recognition deficit. Counteraction of the particular resembling “negative-like” symptom of the cognitive dysfunction was assessed in the novel object recognition test (Figure 1). There were common therapy effects, antagonization (BPC 157), and antagonization (L-arginine). L-NAME, without its own effect, antagonized an L-arginine beneficial effect (NO-system immobilization). On the other hand, BPC 157 overwhelmed the effect of L-arginine as well as the effect of L-NAME. Namely, BPC 157 restated a beneficial effect in the L-NAME+L-arginine rats (NO-system immobilization). With BPC 157 co-administration in the L-NAME+L-arginine+BPC 157, the rats’ beneficial effect reappeared and NO-system immobilization seemed to be counteracted.

**Figure 2:**
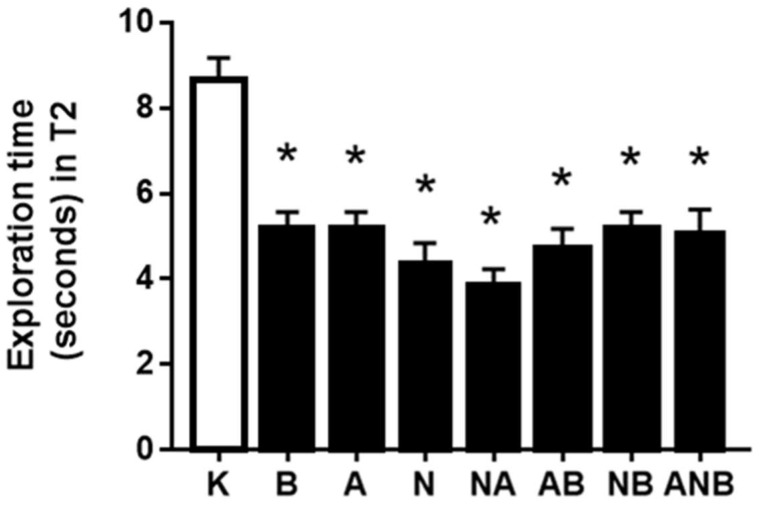
Exploration time expressed by different groups of rats during T2. The exploration time (s) in the novel object recognition test for ketamine-induced disorder in T2, mean values, and 95 percent confidence intervals are presented. The application protocol was injected ip immediately after T1. 1h ITI was used. In ketamine rats (3 mg/kg ip), medication (mg/kg ip) was: L-NAME (5) (N), L-arginine (100) (A), BPC 157 (0.01) (B), alone and/or together (L-NAME+L-arginine (NA), L-arginine+BPC 157 (AB), L-NAME+BPC 157 (NB), L-arginine+L-NAME+BPC 157 (ANB)), and saline 5 mL/kg ip (control ketamine) (K). The application was immediately before ketamine (L-NAME, L-arginine, and combination) or the application was immediately after ketamine (BPC 157 and combinations). Dunnett’s multiple comparison test was used to compare control group (K) with treated groups. * *p* < 0.05 relative to ketamine.

Illustrative for the counteraction of the ketamine-induced cognitive dysfunction, and ketamine-induced prolongation of the exploration time, there was the commonly decreased exploration time spent to recognize (BPC 157, L-arginine) or not recognize (ketamine, L-NAME) the novel object. These were alternative particular parallel effects, i.e., an effect in which L-NAME (antagonization) and L-arginine (antagonization) that could not antagonize each other (Figure 2).

### 3.2. Social Interaction Test

**Figure 3:**
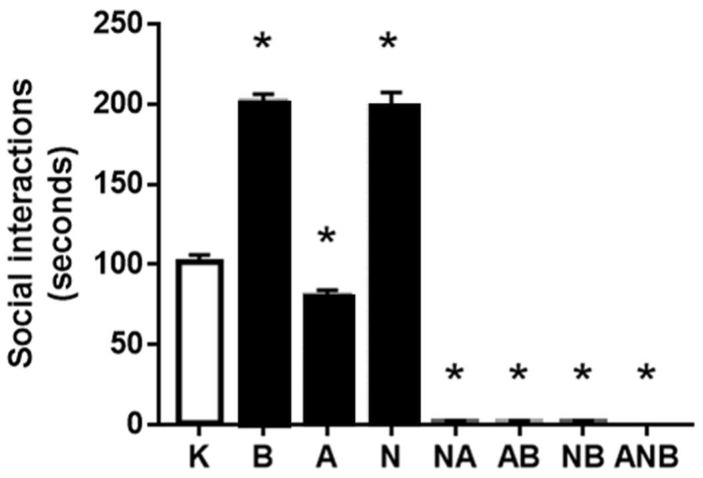
Social interaction. The results for social interaction (s) in open field in the ketamine-induced social interaction test (SIT), mean values, and 95 percent confidence intervals are presented. In the ketamine rats (8 mg/kg ip/3 days), medication (mg/kg ip) was at the last ketamine challenge: L-NAME (5) (N), L-arginine (100) (A), BPC 157 (0.01) (B), alone and/or together (L-NAME+L-arginine (NA), L-arginine+BPC 157 (AB), L-NAME+BPC 157 (NB), L-arginine+L-NAME+BPC 157 (ANB)), and saline 5 mL/kg ip (control ketamine) (K). The application was immediately before ketamine (L-NAME, L-arginine, and combination) or the application was immediately after ketamine (BPC 157 and combinations). Dunnett’s multiple comparison test was used to compare control group (K) with treated groups. * *p* < 0.05 relative to ketamine.

Using the ketamine (8 mg/kg ip) 3 days’ protocol, social withdrawal assessment appeared as “L-NAME responsive, L-arginine non-responsive”. There were common therapy effects, namely antagonization (BPC 157), and antagonization (L-NAME). L-arginine was with opposite (worsening) effect. There was a shared inability of the L-NAME and L-arginine to antagonize each other effect, which may suggest the additional non-NO-system related mechanisms. BPC 157 counteraction potential completely disappeared with L-NAME and L-arginine and combination (Figure 3).

**Figure 4:**
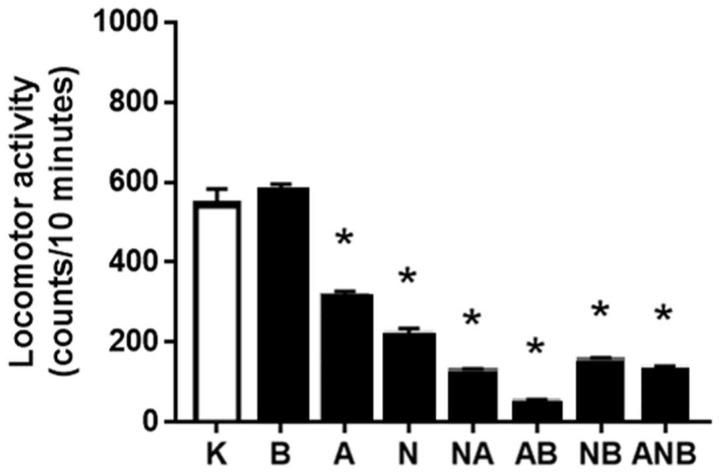
Locomotor activity in social interaction test. The results of locomotor activity in open field in the ketamine-induced social interaction (SIT) test, mean values, and 95 percent confidence intervals are presented. In the ketamine rats (8 mg/kg ip/3 days), medication (mg/kg ip) was at the last ketamine challenge: L-NAME (5) (N), L-arginine (100) (A), BPC 157 (0.01) (B), alone and/or together (L-NAME+L-arginine (NA), L-arginine+BPC 157 (AB), L-NAME+BPC 157 (NB), L-arginine+L-NAME+BPC 157 (ANB)), and saline 5 mL/kg ip (control ketamine) (K). The application was immediately before ketamine (L-NAME, L-arginine, and combination) or the application was immediately after ketamine (BPC 157 and combinations). Dunnett’s multiple comparison test was used to compare control group (K) with treated groups.* *p* < 0.05 relative to ketamine.

Counteraction of the social withdrawal-NO-response described as “L-NAME responsive, L-arginine non-responsive” has a specific point of the counteraction considering the effect on ketamine-locomotor activity. This may be BPC 157 effect (antagonization) as BPC 157 does not affect ketamine (8 mg/kg ip/3 days)-locomotion. Probably fewer NO-system specific effects may be the L-NAME (antagonization) vs. L-arginine (worsening). Namely, both L-NAME and L-arginine reduced ketamine-locomotion. The shared inability of the L-NAME and L-arginine to antagonize each other effect may suggest the additional non-NO-system related mechanisms. In addition, BPC 157 could not overwhelm the effect of NO-agents (Figure 4).

### 3.3. Open Field Test

**Figure 5:**
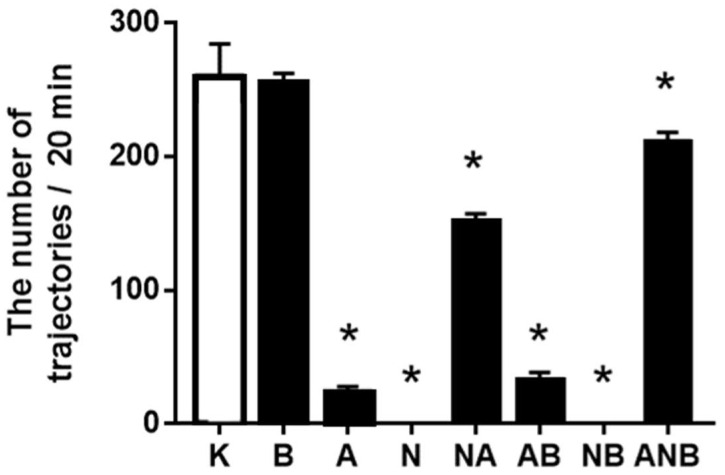
Total number of trajectories in the open field/20 min. The total number of ketamine-induced trajectories in the open field test, mean values, and 95 percent confidence intervals are presented. In the ketamine rats (30 mg/kg ip), medication (mg/kg ip) was: L-NAME (5) (N), L-arginine (100) (A), BPC 157 (0.01) (B), alone and/or together (L-NAME+L-arginine (NA), L-arginine+BPC 157 (AB), L-NAME+BPC 157 (NB), L-arginine+L-NAME+BPC 157 (ANB)), and saline 5 mL/kg ip (control ketamine) (K). The application was immediately before ketamine (L-NAME, L-arginine, and combination) or the application was immediately after ketamine (BPC 157 and combinations). Dunnett’s multiple comparison test was used to compare control group (K) with treated groups.* *p* < 0.05 versus ketamine.

**Figure 6:**
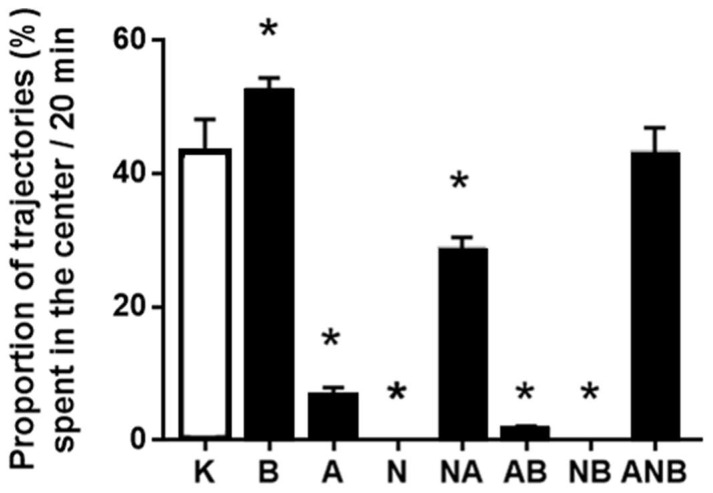
Proportion of trajectories in the center of the open field (%). The proportion of ketamine—induced trajectories in the open field test (OFT), mean values, and 95 percent confidence intervals are shown. In the ketamine rats (30 mg/kg ip), medication (mg/kg ip) was: L-NAME (5) (N), L-arginine (100) (A), BPC 157 (0.01) (B), alone and/or together (L-NAME+L-arginine (NA), L-arginine+BPC 157 (AB), L-NAME+BPC 157 (NB), L-arginine+L-NAME+BPC 157 (ANB)), and saline 5 mL/kg ip (control ketamine) (K). The application was immediately before ketamine (L-NAME, L-arginine, and combination) or the application was immediately after ketamine (BPC 157 and combinations). Dunnett’s multiple comparison test was used to compare control group (K) with treated groups. * *p* < 0.001 relative to ketamine.

BPC 157 did not affect ketamine-locomotion in the open field (Figure 5). This may be a specific effect as BPC 157, known also to have anxiolytic effect, increased the number of the trajectories in the central area (Figure 6). Thus, BPC 157 exerted an additional anxiolytic effect over that anxiolytic effect of ketamine (i.e., compared with the saline-application, ketamine increased the number of the trajectories in the central area (data not specifically shown)).

**Figure 7:**
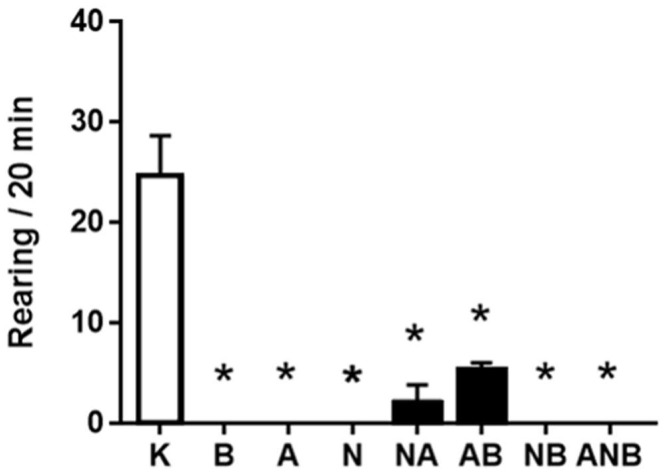
Rearing. The number of rearing induced by ketamine in the open field test (OFT), mean values and 95 percent confidence intervals are shown. In the ketamine rats (30 mg/kg ip), medication (mg/kg ip) was: L-NAME (5) (N), L-arginine (100) (A), BPC 157 (0.01) (B), alone and/or together (L-NAME+L-arginine (NA), L-arginine+BPC 157 (AB), L-NAME+BPC 157 (NB), L-arginine+L-NAME+BPC 157 (ANB)), and saline 5 mL/kg ip (control ketamine) (K). The application was immediately before ketamine (L-NAME, L-arginine, and combination) or the application was immediately after ketamine (BPC 157 and combinations). Dunnett’s multiple comparison test was used to compare control group (K) with treated groups.* *p* < 0.001 relative to ketamine.

NO-agents decreased the number of the trajectories in the central area (Figure 6). Thus, they may have an anxiogenic effect, the parallel worsening L-NAME/L-arginine effect (L-NAME, worsening, L-arginine, worsening) (Figure 6). L-NAME and L-arginine reduced horizontal activity (Figure 6), and reduced vertical activity, reduced rearing number (Figure 7). BPC 157 did not affect horizontal activity (Figure 5), and reduced vertical activity, reduced rearing number, as counteraction of the stimulatory ketamine effect (Figure 7).

### 3.4. Anhedonia

**Figure 8:**
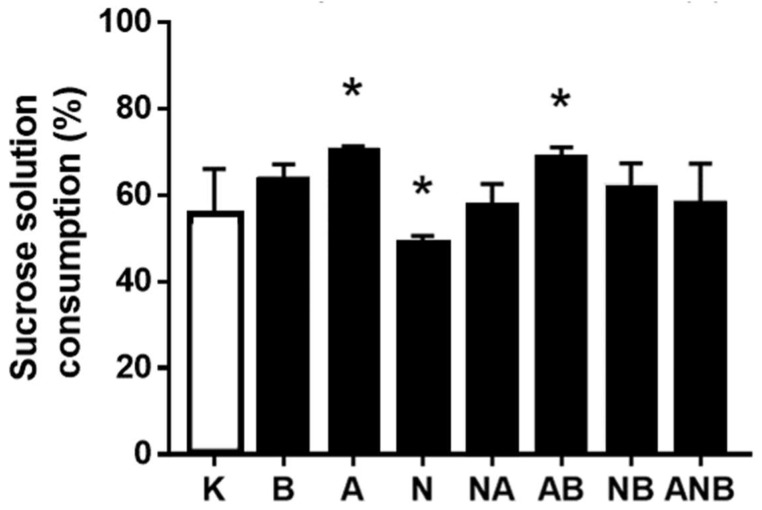
Sucrose solution consumption day 1 (%). Ketamine-induced sucrose consumption is shown in the sucrose preference test (SPT), mean values, and 95 percent confidence intervals. Sucrose preference testing was continued after the open field test (OFT) for 72 h. In the ketamine rats (30 mg/kg ip), medication (mg/kg ip) was: L-NAME (5) (N), L-arginine (100) (A), BPC 157 (0.01) (B), alone and/or together (L-NAME+L-arginine (NA), L-arginine+BPC 157 (AB), L-NAME+BPC 157 (NB), L-arginine+L-NAME+BPC 157 (ANB)), and saline 5 mL/kg ip (control ketamine) (K). The application was immediately before ketamine (L-NAME, L-arginine, and combination) or the application was immediately after ketamine (BPC 157 and combinations). Dunnett’s multiple comparison test was used to compare control group (K) with treated groups. * *p* < 0.05 relative to ketamine.

**Figure 9:**
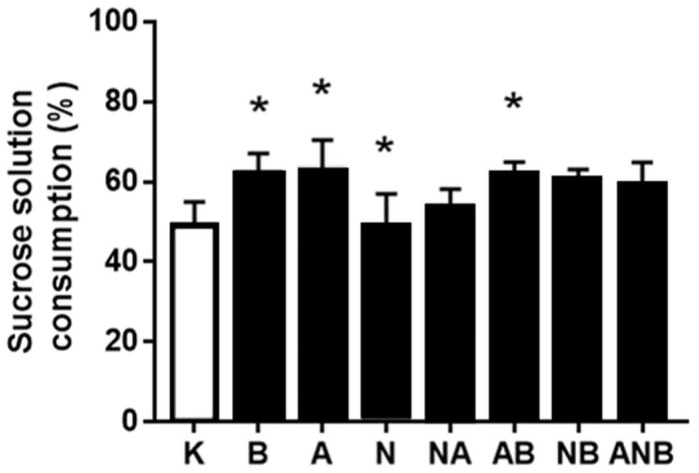
Sucrose solution consumption day 2 (%). Ketamine-induced sucrose consumption is shown in the sucrose preference test (SPT), mean values, and 95 percent confidence intervals. Sucrose preference testing was continued after the open field test (OFT) for 72 h. In the ketamine rats (30 mg/kg ip), medication (mg/kg intraperitoneally) was: L-NAME (5) (N), L-arginine (100) (A), BPC 157 (0.01) (B), alone and/or together (L-NAME+L-arginine (NA), L-arginine+BPC 157 (AB), L-NAME+BPC 157 (NB), L-arginine+L-NAME+BPC 157 (ANB)), and saline 5 mL/kg ip (control ketamine) (K). The application was immediately before ketamine (L-NAME, L-arginine, and combination) or the application was immediately after ketamine (BPC 157 and combinations). Dunnett’s multiple comparison test was used to compare control group (K) with treated groups.* *p* < 0.05 relative to ketamine.

**Figure 10:**
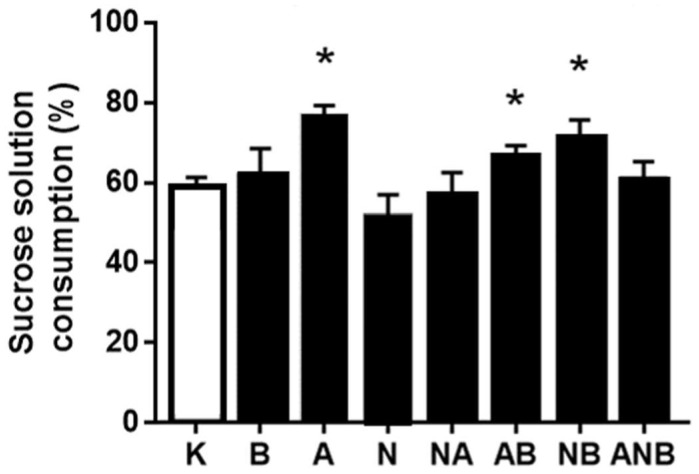
Sucrose solution consumption day 3 (%). Ketamine-induced sucrose consumption is shown in the sucrose preference test (SPT), mean values, and 95 percent confidence intervals. Sucrose preference testing was continued after the open field test (OFT) for 72 h. In the ketamine rats (30 mg/kg ip), medication (mg/kg ip) was: L-NAME (5) (N), L-arginine (100) (A), BPC 157 (0.01) (B), alone and/or together (L-NAME+L-arginine (NA), L-arginine+BPC 157 (AB), L-NAME+BPC 157 (NB), L-arginine+L-NAME+BPC 157 (ANB)), and saline 5 mL/kg ip (control ketamine) (K). The application was immediately before ketamine (L-NAME, L-arginine, and combination) or the application was immediately after ketamine (BPC 157 and combinations). Dunnett’s multiple comparison test was used to compare control group (K) with treated groups. * *p* < 0.05 relative to ketamine.

Considering the antecedent anxiolytic/anxiogenic assessment in the open field test to the sucrose preference test assessment, it may be that “L-NAME responsive, L-arginine responsive” anhedonia NO-response functions (Figure 8, Figure 9 and Figure 10) as a definitive part of the NO-system functioning depending on each of the resembling “negative-like” symptom and given NO-agent(s).

The anhedonia assessment follows after demonstration of the BPC 157 induced anxiolytic effect vs. anxiogenic effect (L-NAME and L-arginine) seeable in the open field test in the ketamine (30 mg/kg ip)-rats, throughout the subsequent three days. BPC 157 (antagonization), L-arginine (antagonization), L-NAME (worsening)) appeared as a particular follow up. The shared ability of the L-NAME and L-arginine to antagonize each other effect went to an inability to antagonize each other effect, and this may indicate the additional non-NO-system related mechanisms. Similarly, considering the counteracting potential ability of the BPC 157 to overwhelm the effect of NO-agents, BPC 157 counteracted L-NAME-effect only.

### 3.5. Gene Expression Analysis

**Figure 11:**
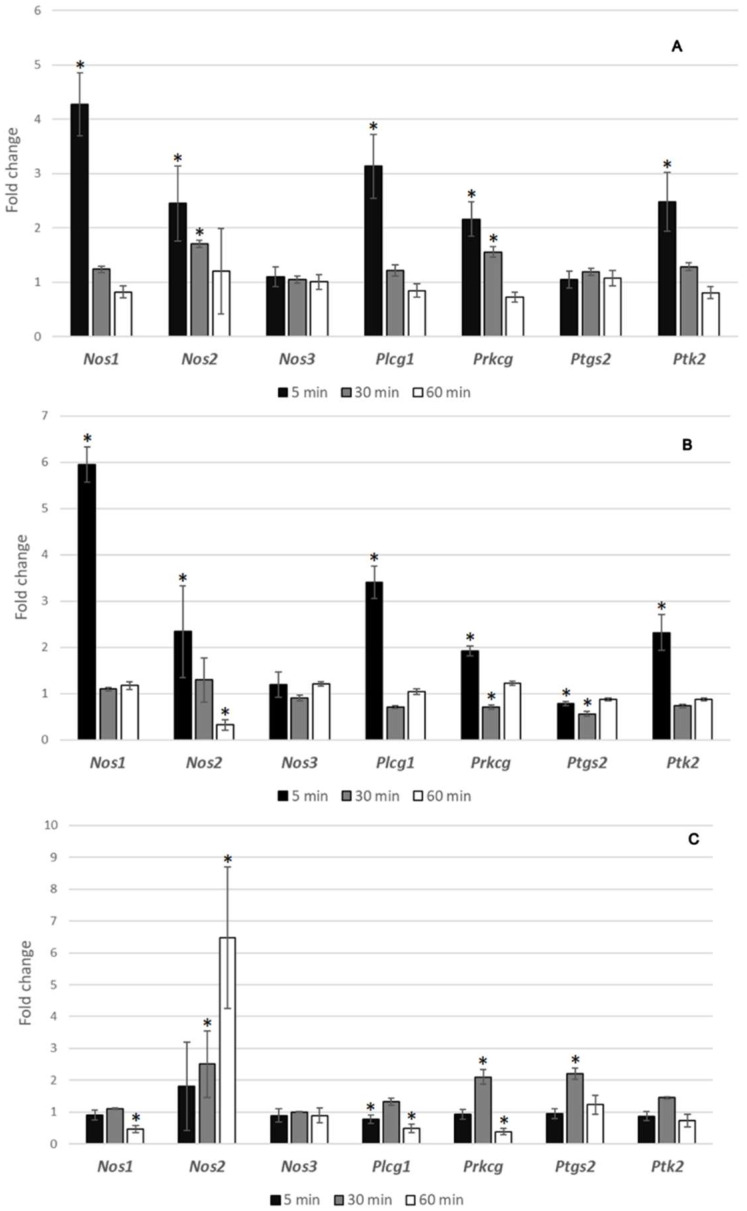
Fold changes in mRNA levels in rat brain samples (**A**–**C**). Healthy rats. (**A**) BPC 157 (10 ng/kg ip) application vs. saline (5 mL/kg ip) application (control); (**B**) Ketamine (30 mg/kg ip) application vs. saline (5 mL/kg ip) application (control); (**C**) Ketamine (30 mg/kg ip)+BPC 157 (10 ng/kg ip) vs. ketamine (30 mg/kg ip)+saline (5 mL/kg ip) (control). Selected genes were tested in three-time intervals: 5, 30 and 60 min. Results are expressed as fold changes: mean ± SD, * marks significant value (*p* ≤ 0.05). Fold change values < 1.00 indicate decreased gene expression in treated animals (downregulation), and fold change values > 1.00 indicate increased gene expression in treated animals (upregulation).

An apparent close connection between the ketamine and BPC 157 was noted in the genes’ expression analysis in brain tissue, using *Nos1*, *Nos2*, *Nos3*, *Plcg1*, *Prkcg*, *Ptgs2*, and *Ptk2* all associated with schizophrenia presentation (Figure 11). We identified the overexpressed genes in the healthy rats treated with the BPC 157 (*Nos1*, *Nos2*, *Plcg1*, *Prkcg*, and *Ptk2*) (Figure 11A) as well as in those treated with the ketamine (*Nos1*, *Nos2*, *Plcg1*, *Prkcg*, *Ptgs2*, and *Ptk2*) (Figure 11B), thus there was a considerable overlapping of gene overexpression, except only to *Ptgs2*. Consequently, the evidenced effect on the given genes’ expression in the brain tissue of the BPC 157 therapy applied immediately after ketamine (i.e., *Nos1* (decreased expression), *Nos2* (increased expression), *Plcg1* (decreased expression), *Prkcg* (increased, and then decreased expression), *Ptgs2* (increased expression), and no effect on *Nos3* and *Ptk2*) (Figure 11C) may be a timely specific BPC 157 effect on ketamine specific targets.

## 4. Discussion

Triple NO-agents application, L-NAME vs. L-arginine vs. combination [8], markedly overwhelmed the regular applicability level in the corresponding NO-studies, achieved with the application of the one single NO-agent (i.e., L-NAME) (i.e., for review, see [3,4]). Thus, triple NO-agents application might consistently combine the ketamine-rats, and the resembling “negative-like” symptoms and the NO-system functions. Consequently, the counteracting effects of the stable pentadecapeptide BPC 157, NO-agents L-NAME and L-arginine might be perceived as mutual interactions put in action or disabled between the NOS-inhibition, NOS-over-stimulation, and NO-system-immobilization. These counteracting interactions might be reliant on the particular agent’s effect, depending on its NO-modulatory ability (BPC 157 > L-arginine > L-NAME antagonizing potential), either able to affect all symptoms in the same way (i.e., BPC 157), or to ameliorate some but worsen others (i.e., L-arginine and L-NAME) [8].

Thus, there was the BPC 157 consistent counteracting potential on the resembling “negative-like” symptoms likely owing its particular interaction with NO-system, and particular NO-modulatory capabilities [8,10] (cognition deficit, social withdrawal, anhedonia and an additional anxiolytic effect).

As before [8], there was less consistent potential of NO-agents, providing that these NO-agents (L-arginine, L-NAME) therapy might be specific NO-therapy, active mostly against a disabled NO-system (L-arginine) or active mostly against over-functioning NO-system (L-NAME) while otherwise being harmful. There were L-arginine-induced counteraction of the cognitive dysfunction, anhedonia, but worsening of the social withdrawal. L-NAME induced counteraction of social withdrawal, but worsening of the cognitive dysfunction and anhedonia. However, an anxiogenic effect shared both L-NAME and L-arginine (and thereby, other systems’ dysfunction additionally involved) [8,10].

In general, for the evidenced BPC 157 > L-arginine > L-NAME antagonizing potential in the resembling “positive-like” symptoms [8], working also with the more complex resembling “negative-like” schizophrenia symptoms, an illustrative example is the counteraction of the cognitive dysfunction, novel object recognition test (Figure 1), as particular resembling “negative-like” symptom [12,13,14]. For the possible but so far not recognized connections, we indicated that this counteraction completely corresponded to the counteracting of the resembling “positive-like” symptoms (acute apomorphine-, chronic methamphetamine-, acute MK-801-induced effects and acute haloperidol-induced catalepsy) [8]. Thus, these might be particular resembling “negative-like” symptoms and particular resembling “positive-like” symptoms that shared the same NO-therapy effect. In “L-NAME non-responsive, L-arginine responsive” NO-response in both circumstances, BPC 157 (antagonization) goes over L-arginine (antagonization). Namely, in all these circumstances (L-NAME-non-responsive), L-NAME antagonized an L-arginine beneficial effect (NO-system immobilization) while BPC 157 co-administration restated a beneficial effect in the L-NAME+L-arginine rats (NO-system immobilization), and thereby, in both circumstances, the L-NAME+L-arginine+BPC 157 rats, BPC 157 counteracted NO-system immobilization [8]. In support of this BPC 157 particular recovering effect on cognitive dysfunction, BPC 157 therapy preserved cognitive function along with the counteracting of the stroke in the rats [33].

Ketamine-prolonged exploration time in the ketamine-induced cognition dysfunction [12,13,14] might be an additional particular matching point (Figure 2). The commonly decreased exploration time spent to recognize (BPC 157, L-arginine) or not recognize (ketamine, L-NAME) the novel object, demonstrated an interesting matching of the alternative particular parallel effects of L-NAME (antagonization) and L-arginine (antagonization) that could not antagonize each other effect, shown in counteraction of the resembling “positive-like” symptoms (acute amphetamine-induced effects) as distinctive NO-system response [8]. Thus, “L-NAME responsive, L-arginine responsive, parallel beneficial effect” NO-response might combine the recovered acute amphetamine-induced effects as the resembling “positive-like” symptom [8] and ketamine-prolonged exploration time in the ketamine-induced cognition dysfunction as the resembling “negative-like” symptom. Besides, NO-system immobilization not achieved (L-NAME and L-arginine did not oppose each other effect) might indicate the essential involvement of other systems as well [8].

Consequently, the newly revealed combined NO-system background (i.e., common therapy effect, common NO-response) [8] might tentatively allocate matching “positive-like” and “negative-like” symptoms or indicate specific circuits between the involved brain areas, mesolimbic pathways in “positive-like” symptoms and fronto-cortical temporal and cortico-striatal pathways in the negative symptoms [1].

It may be that the other resembling “negative-like” symptoms within the used triple application L-NAME–L-arginine–combined L-NAME and L-arginine and stable gastric pentadecapeptide BPC 157 administration did not match with any of the “positive-like” symptoms [8]. Assuming that they occurred also as part of the particular NO-system functioning depending on the each of the resembling “negative-like” symptoms and given NO-agent(s) [8], these additional NO-responses might be distinctive and might occur in a more complex way. “L-NAME responsive, L-arginine non-responsive” (social withdrawal (Figure 3)) NO-response has a specific point of the counteraction, BPC 157 (antagonization) (BPC 157 does not affect ketamine (8 mg/kg ip/3 days)-induced locomotion) (Figure 4), and probably less specific L-NAME (antagonization) vs. L-arginine (worsening) (both L-NAME and L-arginine reduced ketamine-locomotion, and “L-NAME responsive, L-arginine responsive” NO-response in ketamine-locomotion). As pointed out [8], the shared inability of the L-NAME and L-arginine to antagonize each other effect may suggest the additional non-NO-system related mechanisms.

BPC 157 did not affect ketamine (30 mg/kg ip)-locomotion in the open field [18,19,20,21], markedly increased the number of the trajectories in the central area, and thereby, there is an additional anxiolytic effect (for review, see [21,47]) over that anxiolytic effect of ketamine (Figure 5, Figure 6 and Figure 7). Note, anxiolytic effect of ketamine is probably due to ketamine as an antidepressant [48,49] (i.e., compared with the saline-application, ketamine increased the number of the trajectories in the central area (data not specifically shown)). Thereby, considering the BPC 157 general counteracting effect on negative symptoms that might be produced by ketamine administration, the BPC 157 (anxiolytic) effect is particular since it might corroborate with some of ketamine effect as well (for review, see [9,10]). In contrast, with L-NAME or L-arginine, there were in the ketamine rats the worsening, the anxiogenic effect of both L-NAME and L-arginine. These might be also ketamine-specific effect, and thereby particular circumstances aggravation, providing the elevated plus-maze test as an anxiolytic effect of L-arginine [27] as well as L-NAME induced anxiolytic while L-arginine produced anxiogenic effects in the 6-OHDA mouse model of Parkinson’s disease [28]. Consistently in the ketamine-rats treated with either L-arginine or L-NAME, there is the decreased number of the trajectories in the central area, anxiogenic effect, the parallel worsening L-NAME/L-arginine effect (L-NAME, worsening, L-arginine, worsening). Thereby, the NO-response “L-NAME responsive, L-arginine responsive” (anxiogenic effect) appeared as a reversal of the parallel beneficial effect L-NAME/L-arginine effect (L-NAME, antagonization, L-arginine, antagonization) “L-NAME responsive, L-arginine responsive” NO-response in the counteracting “positive-like” symptoms of the acute amphetamine [8]. L-NAME and L-arginine reduced horizontal activity, and reduced vertical activity, reduced rearing number. BPC 157 did not affect horizontal activity, and reduced vertical activity, reduced rearing number, as counteraction of the stimulatory ketamine effect.

Finally, presenting BPC 157 induced anxiolytic effect (for review, see [8,9,10,11]) vs. anxiogenic effect (L-NAME and L-arginine) seeable in the open field test in the ketamine (30 mg/kg ip)-rats, the subsequent three days anhedonia studies (Figure 8, Figure 9 and Figure 10) [17,18] (BPC 157 (antagonization), L-arginine (antagonization), L-NAME (worsening)) appeared as a particular follow up. Thus, it might be that NO-response “L-NAME responsive, L-arginine responsive, opposite effect” (anhedonia) occurred as continuation part of the particular NO-system functioning depending on the each of the resembling “negative-like” symptoms and given NO-agent(s). Again, as mentioned before [8], we can estimate the additional non-NO-system related mechanisms based on the shared inability of the L-NAME and L-arginine to antagonize each other effect (for review, see [8,10]).

The additional non-NO-system related mechanisms as a particular point appeared also in the BPC 157 counteraction in the last resembling “negative-like” symptoms (social withdrawal, anhedonia, anxiogenicity) that seems to be distinctive from the previous one in the resembling “positive-like” symptoms [8] as unable to overwhelm the effects of NO-agents. Unlike its previous wider counteracting potential overwhelming the NO-agents effects shown in all of the “positive-like” symptoms [8] and resembling “negative-like” symptom (cognition deficit), here, BPC 157 counteraction potential completely disappeared with L-NAME and L-arginine. Thereby, i.e., in social dysfunction counteraction, it may be that this BPC 157 beneficial effect is distinctive, but needs intact, not affected NO-system function. Similarly, in the anhedonia (sucrose-test) [17,18], BPC 157 counteracted L-NAME-effect only.

Naturally, the used triple application L-NAME–L-arginine–combined L-NAME and L-arginine and stable gastric pentadecapeptide BPC 157 administration also would need the fMRI and EEG modalities to measure the functional connectivity (i.e., temporal cross-correlations) between brain regions will be preferable to support and understand the presented results in future experiments. Further, atypical antipsychotics [50,51,52], NMDAR indirect agonists [53], NMDAR-glycine site agonists [54] studied in clinical trials for schizophrenia and large list of adjunctive agents with so far little advantage from different classes (i.e., antidepressants [55], psychostimulants [56], anxiolytics [57], anticonvulsants [58], muscarinic receptor agonists and peripehral antagonists [59], selective inhibitor or phosphodiesterase III [60], statins [61]) might also benefit from the studies of the NO-agents triple application and NO-specific therapy relation. Likewise, sampling and analysis of the dopamine, the synaptic/or extra-synaptic GABA, glutamate levels for different brain regions/neuronal circuits during treatments will be helpful. This might be important since ketamine as the model of the schizophrenia (as acute ketamine administration was associated with schizophrenia-like or psychotomimetic symptoms with large effect sizes, increase in positive and negative symptoms [5,6]) might escape from the extraordinary complexity of extrapolation from animal models of mental disorders in general [62].

As a practical contribution to the BPC 157/ketamine relation, we noted a considerable overlapping of gene overexpression as an apparent close connection between the ketamine and BPC 157 (the highest ketamine dose 30 mg/kg ip vs. lowest BPC 157 10 ng/kg ip dose) (Figure 11). This was done with the genes’ expression analysis in brain tissue, using *Nos1* [34,35], *Nos2* [36,37], *Nos3* [38], *Plcg1* [39,40], *Prkcg* [41,42], *Ptgs2* [43], and *Ptk2* [44,45], all associated with schizophrenia presentation. We identified the similar overexpression of the genes in the healthy rats treated with the ketamine (*Nos1*, *Nos2*, *Plcg1*, *Prkcg*, *Ptgs2*, and *Ptk2*) and in the BPC 157 (*Nos1*, *Nos2*, *Plcg1*, *Prkcg*, and *Ptk2*), thus a considerable overlapping of gene overexpression. Thus, the evidenced effect on the given genes expression in the brain tissue of the BPC 157 therapy applied immediately after ketamine (*Nos1* (decreased expression), *Nos2* (increased expression), *Plcg1* (decreased expression), *Prkcg* (increased, and then decreased expression), *Ptgs2* (increased expression), and no effect on *Nos3* and *Ptk2*) may be a timely specific BPC 157 effect on ketamine specific brain targets. Likely, regardless of the possible limitation of results only reflecting mRNA levels, which may not correlate with protein levels [33], this may indicate the way BPC 157, given peripherally, may specifically interfere with the ketamine-induced effects, likely on the specific brain targets. Thus, this might be a direct effect, BPC 157–ketamine. Of note, the previous mRNA expression studies in the stroke rats, at 1 h and 24 h of reperfusion, might be also supportive for an injury specific effect [33]. They provided the full functional recovery (Morris water maze test, inclined beam-walking test, lateral push test), counteracted both early and delayed hippocampal damage, as well as the strongly elevated hippocampal (*Egr1*, *Akt1*, *Kras*, *Src*, *Foxo*, *Srf*, *Vegfr2*, *Nos3*, *Nos1*) and decreased (*Nos2*, *Nfkb*) gene expression (*Mapk1* not activated), as a way how BPC 157 may act [33].

Even before, in the BPC 157/serotonin relations, after peripheral application, particular effect on various brain areas (i.e., especially the serotonin release from the nigrostiratal area) [24] was associated with particular therapy effect, such antidepressant activity (Porsolt’s helplessness) and counteracted serotonin syndrome (for review, see [8,9,10]). Recently, BPC 157, interacting with many molecular pathways [22,23,33,63,64,65,66,67,68,69], was found to act as the membrane stabilizer (counteracted leaky gut syndrome) and free radical scavenger [63]. Note, leaky gut, also takes place in depressed patients and has been related to the inflammatory pathophysiology of the disease [70], as well as leaky gut syndrome, gut permeability may be related to the cognitive and cellular immunity function of patients with schizophrenia [71]. Breakdown of paracellular and vascular pathways and activated neuroimmune and oxidative pathways was established in (deficit) schizophrenia [72]. To this point, as a part of its original cytoprotective capabilities [7], we should mention that BPC 157’s special vascular effect (activation of the collateral pathway) [7] might in a particular way interfere with lithium, known to interact with dopamine and serotonin systems, prototypic agent in bipolar disorder therapy [73]. Recovery of the vascular failure by BPC 157 therapy counteracted lithium-induced multiorgan failure, peripherally and centrally, and lithium-induced particular central and peripheral vascular failure [73]. This might be also interacting with NO-specific molecular pathways [22,23] (i.e., BPC 157 regulates vasomotor tone and the activation of Src-Caveolin-1-endothelial NOS pathway [23]).

Finally, this special issue (i.e., multidimensional NO-dependent interface of schizophrenia modalities presented with triple application of NO-agents, and pentadecapeptide BPC 157, ketamine/BPC 157 relations, and BPC 157, L-arginine and L-NAME therapy potential), remains to be further investigated. Note, the used triple NO-agents application and BPC 157 application as a simple but useful NO-key [8], shared the same dose relation (L-NAME (5 mg/kg), L-arginine (100 mg/kg), BPC 157 (10 µg/kg)) in all BPC 157/NO-studies (for review, see [7,10]). This might be seen as network of the evidence for the physiologic significance of the revealed BPC 157/NO-system interplay (i.e., BPC 157 was found in situ hybridization and immunostaining studies in humans to be largely distributed in tissues [74] and may have additional physiologic regulatory roles [7,74]). Moreover, there is also a very safe BPC 157 profile (i.e., no adverse effects in clinical trials (ulcerative colitis, phase II), and in toxicological studies, lethal dose (LD1) could be not achieved) (for review, see [7,8,9,10,11,74]), a point recently confirmed in a large study conducted by Xu and collaborators [75]. Together, these findings (for review, see [7,8,9,10,11,74]) may be suggestive further BPC 157 therapy application, and appropriate use of the NO-agents in therapy as well.

## Data Availability

The data presented in this study are available on request from the corresponding author.
